# Successful Surgical Treatment of a Ruptured Popliteal Artery Aneurysm With Distal Embolism

**DOI:** 10.7759/cureus.75365

**Published:** 2024-12-09

**Authors:** Yuta Kitagata, Taro Nakatsu, Etsuro Suenaga

**Affiliations:** 1 Cardiovascular Surgery, Kansai Electric Power Hospital, Osaka, JPN

**Keywords:** correct diagnosis, distal embolism, hybrid approach, optimal treatment strategy, popliteal artery aneurysm

## Abstract

A ruptured popliteal artery aneurysm (PPA) is a life-threatening condition that can mimic deep vein thrombosis and lead to critical limb ischemia. Immediate and accurate diagnosis is essential to save the patient’s life and limb. A 73-year-old male presented with acute pain in the posterior aspect of the right knee. Contrast-enhanced computed tomography revealed a ruptured PPA measuring 65 mm with a distal embolism. Emergency surgery was performed, successfully removing the aneurysm and embolus, followed by reconstruction using the saphenous vein. The patient was discharged 12 days postoperatively. This case involved both rupture and embolization, causing hemorrhagic shock and severe leg ischemia. Although endovascular treatment offers outcomes comparable to open surgery in intermediate follow-ups, it may not adequately address severe ischemia in such cases. Open surgical repair with a saphenous vein graft, combined with endovascular techniques, allowed effective bleeding control and restoration of blood flow. This case underscores the importance of rapid diagnosis and tailored treatment strategies to achieve optimal outcomes.

## Introduction

Although popliteal artery aneurysms (PAAs) are the most common type of peripheral artery aneurysm and are known to cause distal embolization and thrombosis, PAA rupture is very rare [1,2]. The optimal treatment method for PAA ruptures differs depending on the patient; therefore, the treatment strategy is complex. Here, we report the successful treatment of a ruptured PAA with distal embolism using a hybrid approach.

## Case presentation

Informed consent was obtained from the patient. This study was approved by the Kansai Electric Power Hospital Institutional Review Board (no. 23-132). The data will be shared by the corresponding author upon reasonable request.

A 73-year-old male patient presented with acute pain in the posterior aspect of the right knee. The patient had no significant medical or family history. On physical examination, his right thigh was swollen, his lower leg was cold, and pulsation of the dorsalis pedis artery and posterior tibial artery made it impossible to measure his blood pressure (162/99 mmHg). His heart rate was irregular at 149 beats/min. Laboratory tests revealed white blood cell, platelet, and hemoglobin counts of 8,500/μL (reference range: 3,500-9,000), 10,0000/μL (reference range: 150,000-40,0000), and 9.4 g/dL (reference range: 14-18), respectively (Table 1).

**Table 1:** Blood test on admission.

	Results	Reference range and units
White blood cell counts	8,500	3,500-9,000 /μL
Platelet counts	100,000	150,000-40,0000 /μL
Hemoglobin counts	9.4	14-18 g/dL

**Figure 1:**
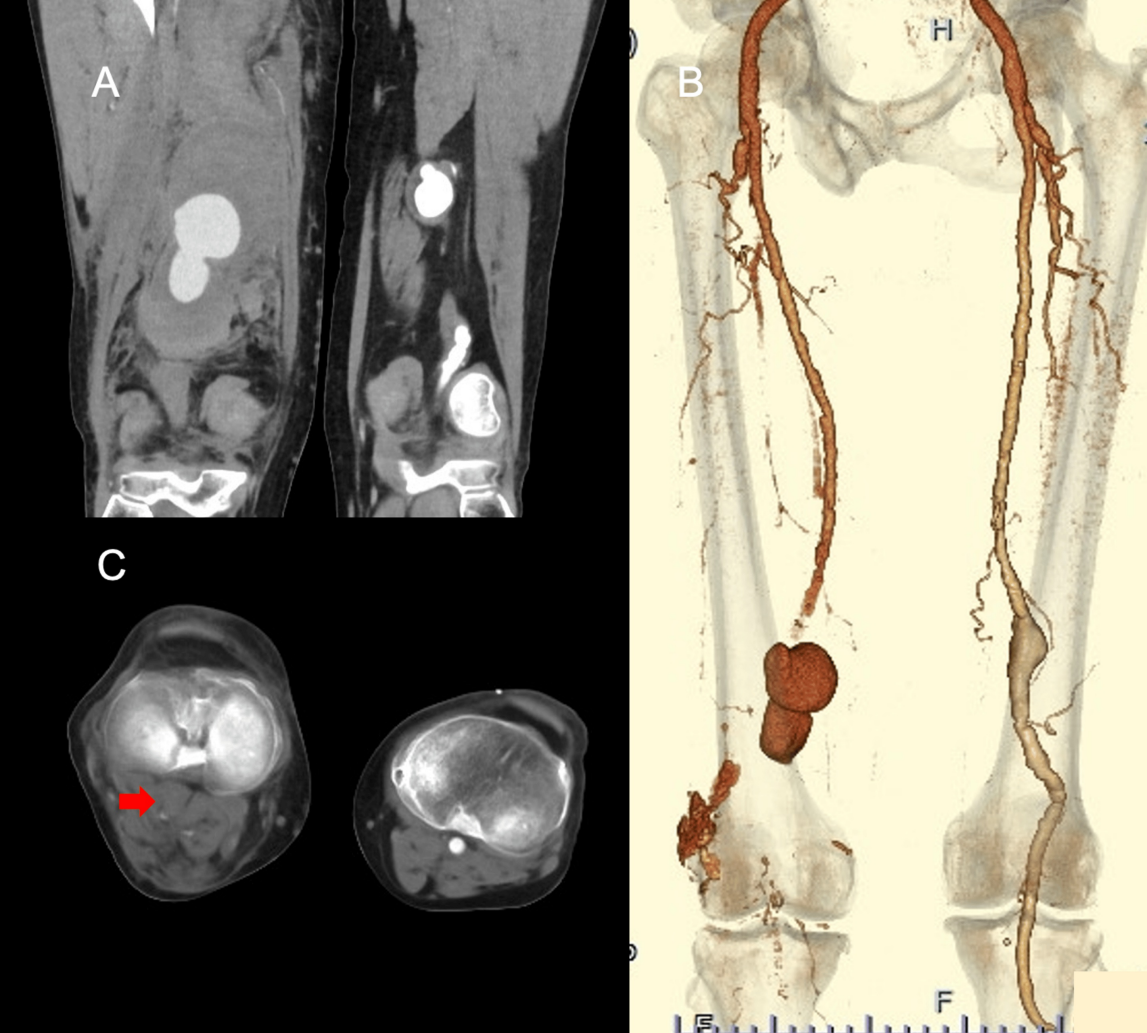
Preoperative computed tomography. (A) Preoperative sagittal enhanced computed tomography showed a ruptured right popliteal artery aneurysm with surrounding hematoma. (B) Preoperative axial enhanced computed tomography showed a popliteal artery distal from the artery without blood flow because of emboli. (C) Preoperative 3D computed tomography indicated that the popliteal aneurysm was located above the knee, with absent distal blood flow. Additionally, there was a right-sided popliteal artery aneurysm.

Contrast-enhanced computed tomography (CT) revealed that both sides of the PPA were affected. The right side of the PAA measured 65 mm and showed evidence of a hematoma around the aneurysm, indicating a rupture (Fig. 1A, 1C). Although blood flow in the lower leg was maintained, there was insufficient collateral blood flow, and the distal popliteal artery was occluded by some embolisms (Fig. 1B).

**Figure 2:**
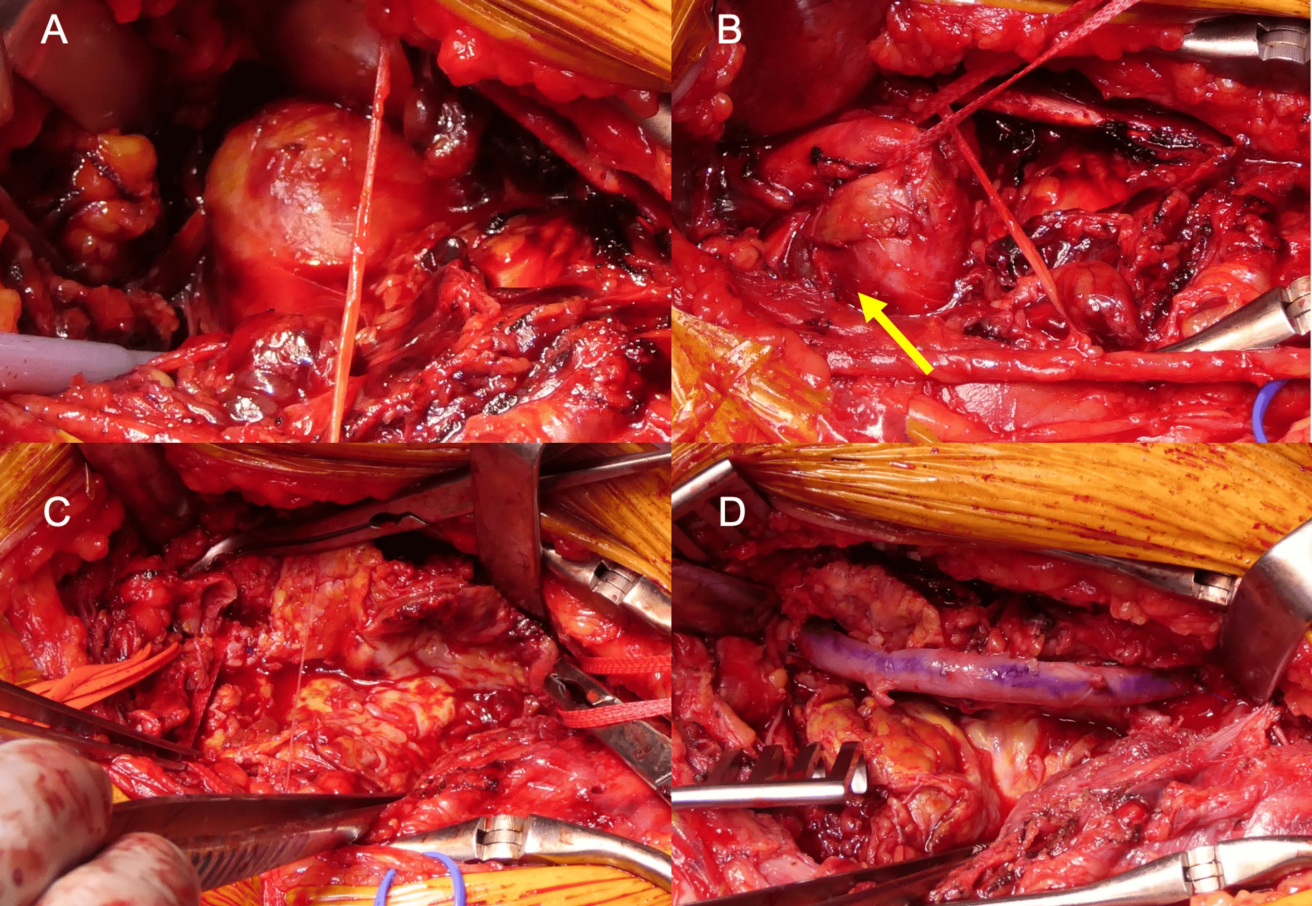
Intraoperative pictures. (A) A giant popliteal aneurysm. (B) The distal popliteal artery without dilation was exposed. (C) The aneurysm was opened, and massive sclerosis was removed. (D) The popliteal artery was reconstructed using an autograft (saphenous vein harvested from the same incision).

A 20-cm skin incision was made on the lower third of the medial thigh above the knee along the anterior margin of the sartorius muscle, and the superficial femoral artery (SFA), which was displaced because of a hematoma, was detected to clamp the blood flow in the mild thigh. After heparization and clamping of the SFA, a PAA with a massive hematoma was detected (Fig. 2A). The rupture site of the PAA was approximately 2 cm proximal to the lower end of the aneurysm (Fig. 2B). No backflow was observed from the distal end of the aneurysm, and the hematoma and atheroma in the aneurysm were removed (Fig. 2C). A 4Fr Fogarty catheter (Edwards Lifesciences, Irvine, CA, USA) was inserted into the distal popliteal artery to pull out the embolisms; upon removal of some of the embolisms and hematomas, backflow was restarted. A great saphenous vein of sufficient size was harvested as a substitute in the same incision and anastomosed edge to edge, proximally, and distally (Fig. 2D). No data indicated that the PAA was infectious.

**Figure 3:**
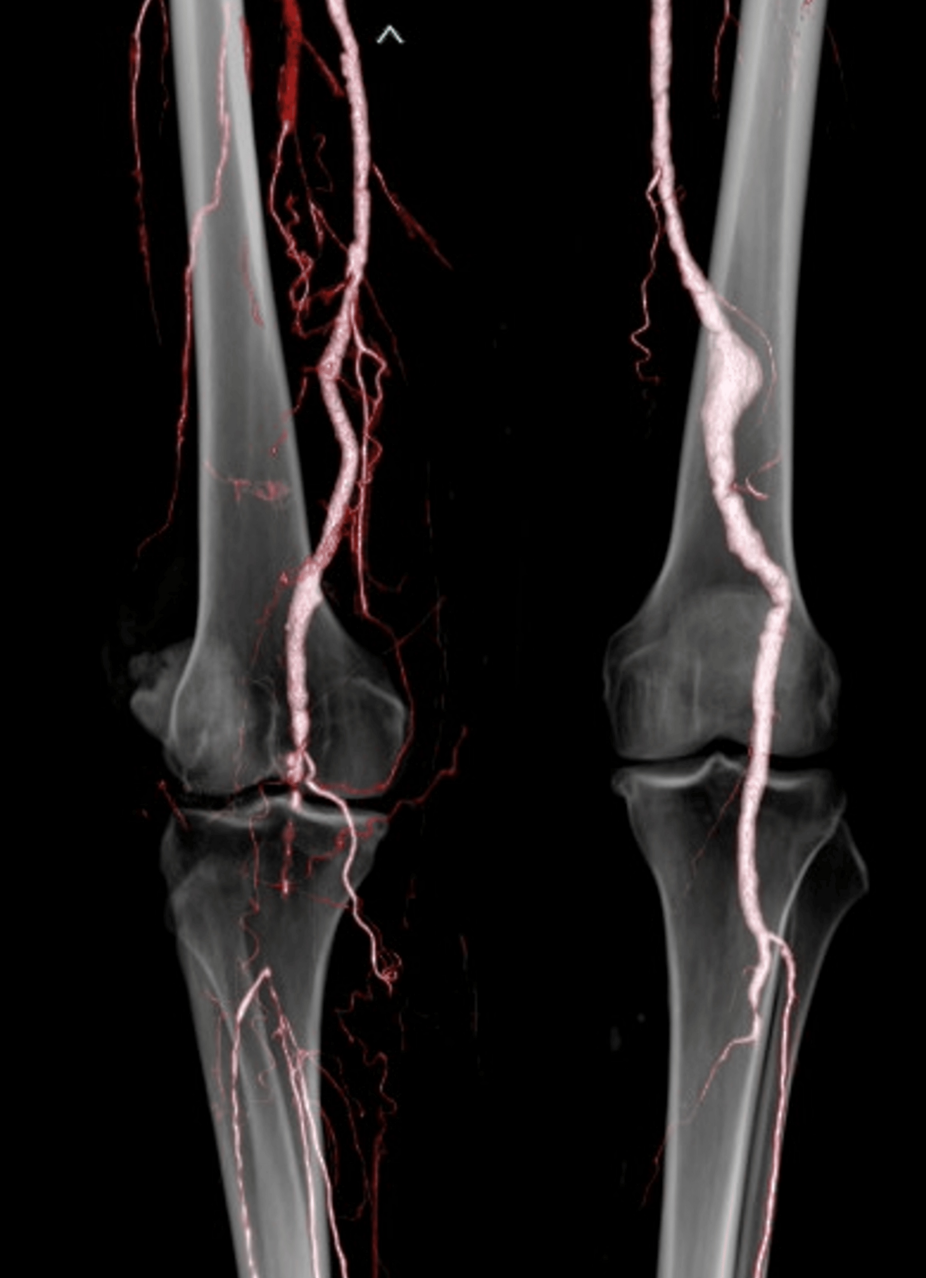
Postoperative computed tomography. Postoperative 3D enhanced computed tomography showed good blood flow in the popliteal artery, with sufficient flow observed below the knee.

The patient was extubated the next day and recovered from hemorrhagic shock. Laboratory tests showed that preoperative creatine kinase levels were within the normal range and did not increase postoperatively. Postoperative contrast-enhanced CT revealed significant blood flow to the lower limbs via the reconstructed popliteal artery, although some small embolisms remained in the distal popliteal artery (Fig. 3). Because the ankle-brachial index improved to 0.76 and no intermittent claudication symptoms were noted, no additional surgical treatment was necessary and the patient could be treated with oral antiplatelet medication. Twelve days after surgery, the patient was discharged.

## Discussion

A ruptured PAA can be life-threatening because it is difficult to diagnose, often mistaken for deep venous thrombosis, resulting in critical limb ischemia [2]. It has been reported that the immediate outcomes after surgery were acceptable; however, there was a high risk of death within one year. Cervin et al. reported an amputation rate of 8.9% and a survival rate of 88.9% after surgery for ruptured PAA [1]. To rescue a patient’s leg and life, a correct diagnosis and appropriate treatment strategy are required.

In the present case, two pathological conditions occurred because of the PAA: rupture and embolization. The rupture caused hemorrhagic shock, and embolization caused severe ischemia in the leg. Some reports on the endovascular treatment of PAAs [3-5] have found that the mid-term results are non-inferior to open surgery [6]. Endovascular treatment may also be considered if treating only the rupture; however, it does not improve the ischemia. In addition to open surgery, it is extremely useful to release the occlusion using endovascular treatment and perform aneurysmal resection and revascularization.

Although most cases are performed by the median approach, it is difficult to clamp the SFA in the prone position. Furthermore, achieving the prone position is difficult when the patient’s vital signs are unstable. In this case, the relative central deviation of the PAA allowed an approach from above the knee and intervention in the peripheral popliteal artery, which made it easier to control the bleeding.

Releasing the occlusion of the distal popliteal artery was essential as blood flow needed to resume as quickly as possible. Using a Fogarty catheter, it was possible to intervene blindly and quickly to remove the peripherally occluded embolus and confirm the resumption of backflow. Postoperative contrast-enhanced CT showed good blood flow to the toes, although some thromboembolisms remained in the popliteal artery, and the ankle blood pressure could be measured. It was thought that there had been some emboli in the popliteal artery prior to this episode, as the preoperative CT showed some distal collateral flow, suggesting significant clonic stenosis of the popliteal artery prior to this episode; only fresh thrombi could be removed and adequate blood flow was restored. Some collateral blood flow to the distal artery developed because of popliteal artery stenosis, which resulted in the maintenance of lower leg blood flow, although the popliteal artery was occluded without elevation of creatine kinase levels after release.

The great saphenous vein was used as a substitute graft because of its thickness. If the vein is too small, it cannot be used. Considering that infection cannot be ruled out as a cause of ruptured aneurysms, they should be treated without artificial vessels, if possible. Owen et al. reported that the outcomes of PAAs did not vary according to the type of graft material used (synthetic or autologous vein) [7]. It has also been reported that prosthetic conduit use in open repair of PAA does not adversely impact outcomes [8]. However, artificial vessels should be used when autologous veins cannot be utilized, especially if infection cannot be ruled out.

## Conclusions

Ruptured PAAs are rare and life-threatening. The treatment strategy is complicated in cases of additional distal embolisms. In the present case, we successfully used surgical and endovascular approaches to treat a ruptured PAA. The optimal strategy was selected based on the most important cases.
